# Comparison of the Psychological Symptoms and Disease-Specific Quality of Life between Early- and Typical-Onset Parkinson's Disease Patients

**DOI:** 10.1155/2014/819260

**Published:** 2014-12-29

**Authors:** Seyed-Mohammad Fereshtehnejad, Hasti Hadizadeh, Farzaneh Farhadi, Gholam Ali Shahidi, Ahmad Delbari, Johan Lökk

**Affiliations:** ^1^Division of Clinical Geriatrics, Department of Neurobiology, Care Sciences, and Society (NVS), Karolinska Institutet, Novum 5th Floor, 14186 Stockholm, Sweden; ^2^Firoozgar Clinical Research Development Center (FCRDC), Firoozgar Hospital, Iran University of Medical Sciences, Tehran 15937-48711, Iran; ^3^Medical Student Research Committee (MSRC), Faculty of Medicine, Iran University of Medical Sciences, Tehran 14496-14535, Iran; ^4^Movement Disorders Clinic, Department of Neurology, Faculty of Medicine, Iran University of Medical Sciences, Tehran 14496-14535, Iran; ^5^Iranian Research Center on Aging, University of Social Welfare and Rehabilitation, Tehran 19857-13834, Iran; ^6^Department of Geriatric Medicine, Karolinska University Hospital, 14186 Stockholm, Sweden

## Abstract

The impact of Parkinson's disease (PD) on psychological status and quality of life (QoL) may vary depending on age of disease onset. The aim of this study was to compare psychological symptoms and disease-specific QoL between early onset versus the rest of the PD patients. A total number of 140 PD patients with the mean current age of 61.3 (SD = 10.4) yr were recruited in this study. PD patients with the onset age of ≤50 yr were defined as “early-onset” (EOPD) group (*n* = 45), while the ones with >50 yr at the time of diagnosis were categorized as the “typical-onset” (TOPD) patients (*n* = 95). Different questionnaires and scales were used for between-group comparisons including PDQ39, HADS (hospital anxiety and depression scale), FSS (fatigue severity scale), MNA (mininutritional assessment), and the UPDRS. Depression score was significantly higher in EOPD group (6.3 (SD = 4.5) versus 4.5 (SD = 4.2), *P* = 0.02). Among different domains of QoL, emotion score was also significantly higher in the EOPD group (32.3 (SD = 21.6) versus 24.4 (SD = 22.7), *P* = 0.05). Our findings showed more severe depression and more impaired emotional domain of QoL in early-onset PD patients. Depression and anxiety play an important role to worsen QoL among both EOPD and TOPD patients, while no interaction was observed in the efficacy of these two psychiatric symptoms and the onset age of PD patients.

## 1. Introduction

Parkinson's disease (PD) is mostly diagnosed in the elderly though a substantial minority of the patients develop symptoms before 50 years of age who are called early-onset Parkinson's disease (EOPD) patients. Patients with EOPD have been detected with rather different characteristics; hence, a different impact of the disease on their lives is expected [1–4]. By developing PD, patients might experience premature aging, increasing dependency, and impairment of occupational performance. The longer the duration of the disease is, the higher physical, economic, and psychological burden of PD often occurs in patients, especially in younger adults with greater socioeconomic responsibilities. On the other hand, treatment-induced motor complications would be higher among the EOPD patients and may contribute to a greater degree of physical impairment and social squeals [2, 3, 5, 6]. Although most of the previous studies have reported poorer quality of life (QoL) in PD patients with earlier age of onset, some findings are still controversial [2, 5, 7–11]. One possible explanation refers to the fact that different motor and nonmotor determinants might affect QoL differently among the EOPD and typical-onset (TOPD) population. More precisely, there are several important variables that were not adjusted between EOPD and TOPD groups in previous studies such as the dosage of levodopa and PD severity.

Growing evidence shows that psychological symptoms and emotional wellbeing play important roles in lowering of QoL in PD patients with earlier disease onset. Therefore, for the sake of better comparison of QoL and its determinants between EOPD and TOPD patients, this study was designed to compare psychological symptoms and disease-specific QoL between EOPD and TOPD patients with similar disease severity measured by scales such as the Hoehn and Yahr stage. Furthermore, a varied list of characteristics were assessed and compared between the two groups including motor and nonmotor symptoms (such as depression and anxiety), fatigue, and nutritional status to understand how differently these factors might influence QoL with regard to the age of disease onset.

## 2. Methods and Materials

### 2.1. Subjects and Setting

A total number of 140 idiopathic Parkinson's disease (IPD) subjects were consecutively recruited from one major outpatient referral Movement Disorder Clinics in Tehran, Iran, during 2011-2012. The study protocol was approved by the Ethics Committee of the Neurology Department at the Firoozgar Clinical Research Development Center (FCRDC) in Tehran, Iran. All collected data was stored and treated according to the ethical guidelines of this research center. Participation in this study was voluntary and each participant was informed about the aims and objectives before the enrollment. The identity of research participants was protected, since the data files were anonymous and all names were omitted.

All cases were enrolled into the study after a thorough physical examination by a neurologist specialized in movement disorders and when the UK brain bank criteria for the diagnosis of IPD [12] were met. Patients with atypical parkinsonism including multiple system atrophy (MSA), progressive supranuclear palsy (PSP), and vascular or drug-induced parkinsonism were not eligible. Moreover, those with moderate to severe dementia (minimental status exam (MMSE) < 24) [13] were also excluded from the study. Using the conventional cut-off value of 50 [2, 14, 15], recruited PD patients with the onset age of ≤50 yr were defined as “*early-onset*” (EO) group (*n* = 45), whereas the ones with >50 yr at the time of diagnosis were categorized as the “*typical-onset*” (TO) PD patients (*n* = 95). Onset age was recorded as the age when PD-related symptoms had firstly started using patients' initial medical records at the clinic.

### 2.2. Assessments

A movement disorder specialist and a group of trained medical students and general physicians performed data collection through interviews and clinical measurements. All clinical assessments were performed during the “On” state and different questionnaires and scales were used to provide data for between-group comparisons. Demographic information consisted of baseline variables, educational status, and comorbidities. PD-related characteristics including disease duration (time passed from diagnosis), measures of disease severity such as the Unified Parkinson's Disease Rating Scale (UPDRS) consisting of four sections on nonmotor symptoms (part I), activities of daily living (ADL) (part II), motor examination (part III) and drug complications (part IV) [16], Hoehn and Yahr stage [17], and Schwab and England activity of daily living (ADL) scale [18] were recorded. Higher scores obtained in UPDRS refer to a more severe condition in PD patients. Current daily dosage of levodopa was measured according to their latest treatment protocol at the time of assessment extracted from patients' medical records. In addition, the weight-adjusted daily dose of levodopa (mg/kg) was calculated as cumulative daily dosage of levodopa in mg divided by body weight in kg for each recruited patient.

We used the validated Persian version of the fatigue severity scale (FSS) [19] in order to measure fatigue severity. This instrument contains nine questions on severity of fatigue during the past week scored from 1 to 7 and a total score, which is obtained by averaging the scores of all items where the higher ones show more severe fatigue [20]. For psychological assessment, the validated Persian version of the Hospital Anxiety and Depression Scale (HADS) was also used [21]. This tool has two sections for depression and anxiety, each of which contains seven questions scored through a range of 0–21 points where higher scores demonstrate worse condition [22]. The Persian version of the Parkinson's disease quality of life questionnaire (PDQ-39) was used to measure health-related quality of life (HRQoL) in PD patients [23, 24]. The PDQ-39 contains 39 Likert-scale items assessing eight domains of disease-related quality of life (QoL) in PD population including mobility, activities of daily living (ADL), emotional wellbeing, stigma, social support, cognitions, communication, and bodily discomfort. The scores range from 0 to 100 and the higher a patient scores, the poorer QoL he/she has experienced [25]. For nutritional status, we used the Mini Nutritional Assessment (MNA) questionnaire comprising 18 items including anthropometric measurements on body mass index (BMI), arm, and calf circumferences. The maximum score in the MNA questionnaire is 30 points, and the higher someone scores, the better nutritional status he/she has [26].

### 2.3. Statistical Analysis

Data were analyzed using SPSS software version 22.0 (IBM., Chicago, IL, USA). In order to describe continuous and qualitative variables, mean (standard deviation (SD)) and frequency (percentage) were used, respectively. The Kolmogorov-Smirnov test was used to check the normality of distribution for numeric variables. In case of skewed distributions, median and interquartile range (IQR) was used for descriptive reports. For univariate comparisons, either independent *t*-test or Mann-Whitney *U*-test was applied to compare the mean values or mean ranks of the normally or nonnormally distributed numeric variables between the two groups, respectively. The Chi square test was performed for between-group comparisons of nominal or categorical variables. Spearman correlation was used to evaluate the linear relationship of the onset age of PD with depression and anxiety scores. Additionally, we applied partial correlation to control the association between the onset age and depression and anxiety by PD duration. For multivariate analysis, linear regression model was applied to assess whether the effect of EOPD on the outcome scores (QoL, depression, anxiety, fatigue, and nutritional status) remains significant after adjustment for potential confounders. Sex, educational level, and comorbidity indicator were selected as well-known confounders to affect psychiatric symptoms and QoL. Although as an intermediary variable, Hoehn and Yahr stage was also included into the model to assess how independent was the effect of EOPD on outcome variables regardless of disease severity as another known determinant factor to influence psychiatric symptoms and QoL in PD. For use in multivariate analysis, a new variable named “comorbidity index” was created by adding up the number of comorbidities each patient had consisting of hypertension, interstitial heart disease, stroke or transient ischemic attack, diabetes, osteoarthritis, and chronic obstructive pulmonary disease. In all analytical procedures, a two-sided *P* value of <0.05 was considered as the statistical significant level to reject the corresponding null hypothesis.

## 3. Results

### 3.1. Demographic Characteristics

Of 140 patients who completed the study, 45 had onset of PD before age 50 (EOPD) and 95 had onset age after 50 years (TOPD). The average current age of the whole study population was 61.3 (SD = 10.4) yr and the range for age of onset was 20–50 years in the EOPD and 51–77 years in the TOPD group.  Table 1 shows their baseline, clinical, and sociodemographic characteristics. The male preponderance was higher in the TOPD group (2.39) compared to that of the EOPD (1.65); however, sex distribution was not significantly different between the two groups (*P* = 0.326). As expected, duration of PD was significantly longer among the EOPD patients [8.5 (SD = 7.7) versus 5.9 (SD = 3.7), *P* = 0.035]. Nevertheless, no significant difference was found in the severity of PD assessing by neither the Hoehn and Yahr stage (EOPD 2.0 (IQR = 2.0) versus TOPD 2.0 (IQR = 1.5), *P* = 0.923), nor the Schwab and England ADL score (EOPD 80.4% (SD = 19.6) versus TOPD 82.4% (SD = 15.1), *P* = 0.553) and nor the total UPDRS score (EOPD 34.3 (SD = 21.0) versus TOPD 30.6 (SD = 15.2), *P* = 0.326). The EOPD patients received a significantly higher daily dosage of levodopa (1000 (IQR = 710) mg versus 750 (IQR = 500) mg, *P* = 0.030) even after weight-adjustment (13.4 (IQR = 12.2) mg/kg versus 10.4 (IQR = 6.7) mg/kg, *P* = 0.040).

### 3.2. Psychiatric Features

Table 2 summarizes the results for comparing anxiety, depression, and fatigue scores between the two study groups. Depression score was significantly higher among the EOPD patients (6.3 (SD = 4.5) versus 4.5 (SD = 4.2), *P* = 0.02), while anxiety and fatigue scores failed to show any significant difference between the two groups. Based on the cutoff value of ≥8 for the HADS scores [27], the prevalence of depression was significantly higher in the EOPD group (40% versus 21%, OR = 2.5 (95% CI 1.2 to 5.4); *P* = 0.019). A higher proportion of anxious patients were seen in the EOPD group (47% versus 35%, OR = 1.6 (95% CI 0.8 to 3.4); *P* = 0.176); however, the difference was not statistically significant.

**Figure 1:**
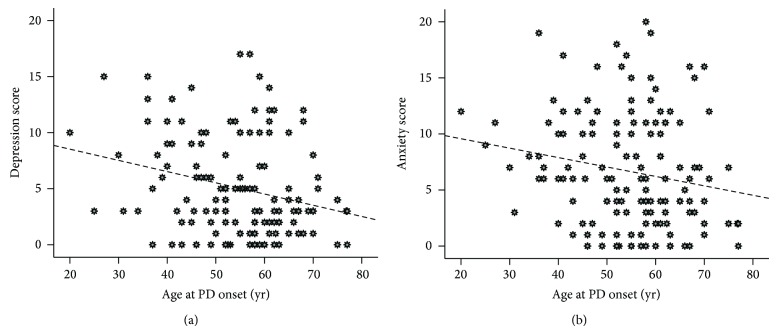
Linear correlation of the age of disease onset with (a) depression and (b) anxiety scores among the subjects with idiopathic Parkinson's disease.

As shown in Figure 1, Spearman correlation statistic showed a significant inverse correlation between the age at PD onset and both depression (Spearman rho = −0.226, *P* = 0.007) and anxiety (Spearman rho = −0.171, *P* = 0.043) scores. The results of partial correlation demonstrated that these associations remained statistically significant even after controlling for the duration of PD (for depression: *r* = −0.214, *P* = 0.011; for anxiety: *r* = −0.167, *P* = 0.049). Fatigue score was not correlated with the onset age of PD both before and after adjustment for disease duration (*P* = 0.279 and 0.594, resp.). As shown in Table 2, multivariate adjustment was performed to compare the comprehensive list of psychiatric symptoms and fatigue between the two study groups. Multivariate linear regression modeling demonstrated that the EOPD patients had approximately 1.4 unit higher depression score even after adjustment for sex, education level, comorbidity index, and the Hoehn and Yahr stage as the possible proxy for disease severity (*B* = 1.42 (95% CI: −0.01 to 2.85), *P* = 0.051). However, other outcomes failed to show any significant difference between the EOPD and TOPD patients after multivariate adjustments (all *P* > 0.05).

### 3.3. Health-Related Quality of Life (HRQoL)

The scores for different domains of the PDQ-39 questionnaire and the MNA scale for nutrition are listed in Table 2. The total score of the PDQ-39 (EOPD 21.9 (SD = 14.6) versus TOPD 19.0 (SD = 13.5), *P* = 0.25) and the MNA (EOPD 24.4 (SD = 4.3) versus TOPD 25.5 (SD = 2.8), *P* = 0.07) was not significantly different between the two study groups. However, the EOPD patients had significantly worse emotional wellbeing compared to the TOPD group (EOPD 32.3 (SD = 21.6) versus TOPD 24.4 (SD = 22.7), *P* = 0.05). Further multivariate linear regression analysis was performed to assess the factors that affect HRQoL (total score of the PDQ-39) and their interactions with study groups (EOPD versus TOPD). As shown in Table 3, EOPD was not an independent determinant factor for QoL in PD population (*P* = 0.815). Anxiety (*B* = 0.6 (95% CI: 0.2–1.0), *P* = 0.003) and depression (*B* = 1.5 (95% CI: 0.9–2.0), *P* < 0.001) significantly affected QoL in entire PD population, while no significant interaction was found between psychiatric symptoms, fatigue and nutritional score with the grouping variable (EOPD versus TOPD) in their effects on QoL score.

Sensitivity analysis was performed to check the variability of the findings when the cutoff point for onset age was changed. Neither lowering the cutoff value for the onset age to 45 yr (EOPD (*n* = 28): 23.1 (SD = 15.3) versus TOPD (*n* = 112): 19.2 (SD = 13.5), *P* = 0.179) nor increasing it to 55 yr (EOPD (*n* = 71): 21.9 (SD = 14.4) versus TOPD (*n* = 69): 18.0 (SD = 13.1), *P* = 0.100) did result in significant difference in the total HRQoL score between the two study groups. Multivariate linear regression analysis was also rerun while a younger age of 45 yr was selected to define EOPD, which showed no significant effect for the grouping variable (EOPD versus TOPD) on the HRQoL score (*B* = 2.8 (95% CI: −30.2–35.8), *P* = 0.867). In contrast, when the cutoff age was moved to a higher value of 55 yr, the new defined EOPD showed significantly lower PDQ-39 score in the same multivariate regression model (*B* = −35.8 (95% CI: −68.9–−2.7), *P* = 0.034).

## 4. Discussion

Our results suggest that patients with EOPD experience worse condition in some psychological features mainly depression compared to the TOPD group. While disease duration was significantly longer and daily dosage of levodopa received by patients was higher among the EOPD group, disease severity did not significantly differ between the two groups which further highlights the importance of the independent psychosocial burden in PD. However, this nonsignificant difference in PD severity could be also due to the rather small sample size in each study group, too. In line with previous studies depression score was significantly higher among the EOPD patients [2, 7, 28–30]. Besides, our study demonstrates that this difference remains significant, although statistically borderline, after multivariate adjustment for sex, education level, comorbidity burden, and the Hoehn and Yahr stage. This highlights the role of psychosocial factors apart from the motor features of the disease, including role expectations. Schrag et al. suggest that older patients are more compatible with physical impairments and employment issues such as early retirement [2]. Findings from another study by Starkstein et al. suggest that EOPD patients have higher frequency of depression and their depression associates with cognitive impairments while late-onset patients showed lower frequency of depression which was mainly associated with activities of daily living [29]. We showed that anxiety score had an inverse correlation with the onset age of PD; however, the difference among two groups did not reach significant level as also showed by Schrag et al. [2]. Our study is one of the few to evaluate the correlation between fatigue score and onset age of PD, which was not statistically significant. Nonetheless, Shulman et al. declared that more than half of the times, presence of depression, anxiety, and fatigue is not even identified [31].

We assessed health-related quality of life using the PDQ-39 and nutritional status by means of the MNA scores, none of which was significantly different between EOPD and TOPD groups. Two other studies done by Knipe et al. [7] and Schrag et al. [2] showed that total score of PDQ-39 was significantly worse in young-onset patients. More detailed comparison reveals that Knipe et al. [7] have used the cutoff age of 45 yr to define EOPD group. In our sensitivity analysis, the cutoff value of 45 yr for definition of EOPD also failed to show any significant independent effect on QoL. We must also acknowledge the higher sample size in the similar study performed by Knipe et al. [7] that had higher statistical power to show small differences in QoL scores between the EOPD and TOPD groups. However, similar to Knipe et al. [7] we also demonstrated that the score on* “emotional wellbeing”* as a domain of the PDQ-39 was significantly poorer in EOPD patients. This further highlights the importance of the psychological aspects of PD among the younger patients. Knipe et al. suggested that the difference in quality of life between EOPD versus TOPD patients could originate from the higher levels of depressive mood in the former group [7]. Results from multivariate regression analysis in our study showed that psychiatric features, namely, depression and anxiety, were the factors that mainly affected QoL in the whole PD population. This association, however, was found to be independent of onset-age and the interaction terms between depression, anxiety, fatigue, and nutritional status with EOPD/TOPD were not significant. In other words, depression and anxiety play a considerable role to worsen QoL among both EOPD and TOPD patients.

Our study is one of the few attempts to compare a comprehensive list of symptoms and conditions between the EOPD and TOPD patients with a fairly deeper exploration of the determinant factors of QoL in each subgroup. Our study has described a previously unstudied population of Iranian people with PD using validated Persian versions of assessment scales. Moreover, we were able to include a good representation of different ages and genders in this PD population. As another point of interest, the levodopa cumulative dose in our study refers only to L-Dopa itself and not the other dopaminergic drugs and is not confounded by the agonists' effect on mood. Yet there are some limitations as well including the rather small sample size and cross-sectional design of the study. Moreover, disease duration was significantly different which might have affected psychosocial aspects of the disease. We tried partial correlation analysis in order to adjust potential confounders though. Having recruited patients from an outpatient tertiary clinic might have induced a selection bias through which undiagnosed patients are missed (potentially those with lower disease severity), as well as those end-stage hospitalized PD patients. This should be considered while generalizing our findings.

In conclusion, our findings draw more attention to the important phenotypic differences between the EOPD and TOPD patients. Since depression is more common in younger PD patients which consequently affects QoL, its screening and further interventions seem necessary. Depression and anxiety found to be the most important determinants of QoL in both EOPD and TOPD patients. Physicians need to focus more on nonmotor symptoms of PD including depression and anxiety that mainly worsen QoL. Accurate and timely diagnosis of depression and anxiety is crucial to improve QoL in PD patients as early as possible since these symptoms could be even more frequently seen in younger PD patients. Besides, it seems rational that people who live with PD patients such as their caregivers become informed about depressive symptoms and try to diminish psychosocial stress and burden as well.

## Figures and Tables

**Table 1:** Baseline, clinical, and sociodemographic characteristics of the early-onset and typical-onset Parkinson's disease patients.

Characteristics	Early onset (*n* = 45)	Typical onset (*n* = 95)	*P* value
Gender no. (%)			
Female	17 (37.8)	28 (29.5)	0.326
Male	28 (62.2)	67 (70.5)	
Level of education^**^ no. (%)			
Illiterate	2 (4.7)	10 (10.5)	0.137
Primary and/or secondary	11 (25.6)	24 (25.3)	
High School/Diploma	17 (39.5)	21 (22.1)	
College and/or University	13 (30.2)	40 (42.1)	
Duration of disease (yr)			
Mean (SD)	8.5 (7.7)	5.9 (3.7)	0.035^*^
UPDRS score mean (SD)			
Part I-mental	2.3 (2.8)	1.8 (1.9)	0.407
Part II-ADL	12.5 (7.6)	10.6 (6.8)	0.148
Part III-motor	15.7 (11.5)	15.2 (7.6)	0.490
Part IV-complications	4.0 (3.4)	3.3 (2.4)	0.198
Total	**34.3** (**21.0**)	**30.6** (**15.2**)	**0.326**
Hoehn and Yahr Stage			
Median (IQR)	2.0 (2.0)	2.0 (1.5)	0.923
Schwab and England activities of daily Living Score (%)			
Mean (SD)	80.4 (19.6)	82.4 (15.1)	0.553
Levodopa dose (mg) median (IQR)			
Cumulative Daily Dose	1000.0 (710.0)	750.0 (500.0)	0.030^*^
Weight-adjusted daily dose	13.4 (12.2)	10.4 (6.7)	0.040^*^

^*^Difference is statistically significant at the 0.05 level (2-tailed).

^**^Data on education level is not available for two patients (missing value) and valid relative percentages are reported.

**Table 2:** Univariate and multivariate comparison of the mean (standard deviation (SD)) scores of different motor, nonmotor, and quality of life (PDQ-39) scales between subgroups of Parkinson's disease (PD) patients regarding the age of disease onset.

Scale	Domain	Early-onset (*n* = 45)	Typical-onset (*n* = 95)	Univariate comparison		Multivariate comparison	
				Unadjusted *B* (95% CI)	*P* value	Adjusted *B* (95% CI)	*P* value
PDQ39	Mobility	26.6 (26.8)	26.9 (24.5)	−0.3 (−9.4–8.7)	0.94	−2.1 (−9.4–5.3)	0.58
	Activities of daily living (ADL)	24.8 (25.0)	21.9 (20.6)	2.9 (−5.2–11.0)	0.48	1.8 (−5.3–8.9)	0.61
	Emotional wellbeing	32.3 (21.6)	24.4 (22.7)	7.9 (−0.1–15.9)	0.05^*^	6.3 (−1.3–14.0)	0.11
	Stigma	25.1 (26.9)	19.8 (24.6)	5.3 (−3.7–14.4)	0.25	5.6 (−4.0–15.1)	0.25
	Social support	9.6 (15.7)	6.2 (12.2)	3.3 (−1.8–8.4)	0.20	0.2 (−4.4–4.9)	0.93
	Cognitive impairment	16.7 (18.1)	16.8 (17.9)	−0.1 (−6.5–6.3)	0.97	0.1 (−6.4–6.5)	0.99
	Communication	18.0 (22.6)	11.9 (14.1)	6.0 (−0.1–12.2)	0.06	3.2 (−2.4–8.8)	0.26
	Bodily discomfort	20.4 (20.2)	20.7 (21.6)	−0.3 (−7.9–7.3)	0.93	−1.9 (−9.0–5.2)	0.60
	Total	**21.9** (**14.6**)	**19.0** (**13.5**)	**2.9** (−**2.1**–**7.8**)	**0.25**	**1.6** (−**2.8**–**6.0**)	**0.49**

HADS	Anxiety	7.5 (4.7)	6.3 (5.3)	1.2 (−0.6–3.1)	0.18	1.2 (−0.6–3.0)	0.20
	Depression	6.3 (4.5)	4.5 (4.2)	1.8 (0.3–3.3)	0.02^*^	1.4 (0–2.9)	0.05^*^

FSS	Fatigue	4.7 (1.8)	4.4 (1.9)	2.8 (−3.4–8.9)	0.38	2.2 (−3.5–8.0)	0.48

MNA	Nutritional status	24.4 (4.3)	25.5 (2.8)	−1.1 (−2.3–0.1)	0.07	−0.9 (−2.0–0.2)	0.10

CI: confidence interval.

^*^Difference is statistically significant at the 0.05 level (2-tailed).

(Multivariate adjustment has been performed considering sex, education level, comorbidity index, and Hoehn and Yahr stage as potential confounders).

**Table 3:** Multivariate linear regression model for the effect of EOPD on health-related quality of life (QoL) score and its interactions with other determinant factors.

Scales/Variables	Coefficients		Wald Chi^2^	95% CI for *B*		*P* value
	*B*	SEM		Lower bound	Upper bound	
EOPD	−3.7	15.7	0.1	−34.3	27.1	0.814
Female sex	2.9	1.7	3.0	−0.4	6.2	0.082
Education level						
Illiterate	0 (ref.)	—	—	—	—	—
Primary and/or Secondary	−3.6	2.8	1.6	−9.1	2.0	0.208
High School/Diploma	−2.8	2.9	1.0	−8.4	2.8	0.323
College and/or University	−3.8	2.8	1.8	−9.3	1.7	0.176
Comorbidity index	0.2	0.8	0.1	−1.4	1.8	0.812
Hoehn & Yahr stage	0.8	0.9	0.8	−1.0	2.7	0.384
Nutritional status (MNA score)	−0.5	0.4	1.8	−1.3	0.2	0.184
Interaction with EOPD	0.1	0.5	0.0	−0.9	1.2	0.829
Anxiety (HADS score)	0.6	0.2	8.9	0.2	1.0	0.003^*^
Interaction with EOPD	0.2	0.4	0.2	−0.6	0.9	0.695
Depression (HADS score)	1.5	0.3	26.7	0.9	2.0	<**0.00**1^*^
Interaction with EOPD	−0.5	0.5	0.9	−1.5	0.5	0.344
Fatigue (FSS score)	0.1	0.1	3.3	−0.0	0.2	0.071
Interaction with EOPD	0.0	0.1	0.0	−0.2	0.2	0.846

^*^Statistically significant factor at the significance level of 0.05 (2-tailed).

(Multivariate adjustment has been performed considering sex, education level, comorbidity index, and Hoehn and Yahr stage as potential confounders).
